# Can MRI knee joint measurements predict the population at risk of ACL injury?

**DOI:** 10.1186/s13102-022-00495-1

**Published:** 2022-06-02

**Authors:** Mohammad Hamdan, Bassem Haddad, Mohammad Ali Alshrouf, Muayad I. Azzam, Ula Isleem, Reem Hamasha, Omar M. Albtoush, Muna Tayel Alhusban, Nidaa Mubarak, Saif Aldeen Alryalat

**Affiliations:** 1 Division of Orthopaedics, Department of Special Surgery, School of Medicine, The University of Jordan, Amman, Jordan; 2 The School of Medicine, The University of Jordan, Queen Rania Street, Amman, 11942 Jordan; 3 Department of Orthopedic Surgery, Icahn School of Medicine at Mount Sinai, New York, NY USA; 4 Department of Radiology, University of Jordan, Amman, Jordan; 5 Department of Special Surgery, Division of Ophthalmology, The University of Jordan, Amman, Jordan

**Keywords:** Anterior cruciate ligament, ACL tears, Risk factor, Knee morphology, Magnetic resonance imaging

## Abstract

**Background:**

Anterior cruciate ligament (ACL) injuries have been increasing significantly over time. The relationship between the ACL injury and the knee joint structures is poorly understood. The purpose of this study is to examine whether the measurements of different structures in the knee joint are linked with ACL injury in affected patients.

**Methods:**

This retrospective case–control study included patients who suffered from ACL tears and underwent magnetic resonance imaging (MRI). A control group of patients with no knee pathologies on MRI was included. Fourteen knee variables, including lateral meniscus (LM) posterior horn height, length, depth, and volume; medial meniscus (MM) posterior horn height, length, depth, and volume; lateral and medial (MFC) femoral condyle sphere diameter; lateral and medial tibial plateau length; and patella tendon horizontal and vertical diameter, were collected. A multivariate logistic regression including LM posterior horn depth, MM posterior horn length, MM volume, MFC sphere diameter, and patella tendon horizontal diameter and receiver operating characteristic curve, was used to compare the two groups.

**Results:**

A total of 85 patients were included in our study; 54 suffered from ACL injuries and 31 as a control group with normal knee MRI. Logistic regression revealed that increased LM posterior horn depth (OR = 1.27; 95% CI = 1.03–1.56; *p* = 0.028), decreased MM posterior horn length (OR = 0.71; 95% CI = 0.55–0.93; *p* = 0.013), and MFC sphere diameter (OR = 1.20; 95% CI = 1.01–1.43; *p* = 0.035) were independent risk factors for ACL rupture. The MFC sphere diameter yielded the highest area under the curve: 0.747 (95% CI, 0.632–0.862). No difference was found in the other measurements between the two groups.

**Conclusions:**

Concerning the difference in anatomical variations, the lateral meniscus posterior horn depth and medial femoral condyle sphere diameter were higher, while medial meniscus posterior horn length was lower in patients with an ACL injury. These structural knee measurements could have a possible increase in the likelihood of sustaining an ACL injury and can be used by clinicians to predict ACL injury.

## Introduction

The anterior cruciate ligament (ACL) is a knee ligament that runs from the medial aspect of the lateral femoral condyle through the intercondylar fossa to the medial tibial eminence [1]. Magnetic resonance imaging (MRI) is the ideal imaging method for knee evaluation and ACL injuries, which has been shown to have high sensitivity and specificity [2]. The ACL is blended with the anterior horn of the medial meniscus anterior to the intercondylar eminence and runs from 11 mm in width to 17 mm anteroposteriorly [3]. The function of the ACL is the containment of anterior translation, primarily. However, the ACL also prevents lateral rotation, varus and valgus stress, extension, and hypertension [1].

The ACL is the most commonly damaged ligament in the knee, with 100,000 to 200,000 injuries occurring each year in the United States [4]. Sports and sports-related activities account for the majority of ACL injuries [5]. This injury disproportionately affects the younger population, particularly teenagers and college-aged adults, and has been steadily increasing due to increased interest in organized sports, particularly among females [6]. This can have a negative psychological and physical impact on them [7], and may even cause them to retire from the sport sooner than they would have otherwise [8]. In addition, it might increase the risk of early-onset post-traumatic osteoarthritis and functional limitation regardless of the treatment administered [9].

Approximately three-quarters are non-contact injuries, highlighting the need to identify risk factors in order to target prevention strategies [10]. Several risk factors have been investigated in an attempt to reduce the risk of ACL injury, including female gender, genetic predisposition, hormonal levels, prior reconstruction of the ACL, lower extremity biomechanics, and impaired neuromuscular control [6, 11–13]. Therefore, preventative programs for ACL injuries have been incorporated in recent years and demonstrated to lower injury rates, especially focusing on modifiable risk factors in at-risk populations, which has been shown to be a cost-effective strategy [13–15].

Few studies have examined the correlation between ACL injury and the anatomical variations found in every individual, where several anatomical structures have been identified as a risk factor for ACL injury [16–20]. Therefore, it is of great importance to study the difference in anatomical variations between patients with an ACL injury and healthy individuals that might help implement appropriate techniques or changes in the lifestyle of individuals, particularly those who participate in high-risk sports. Thus, this study aims to examine the measurements of different anatomical structures in the knee joint between ACL-injured and non-injured patients and how these measurements correlate with an ACL injury in affected patients.

## Material and methods

### Study design

A retrospective case–control study was carried out over a period of 5 years, from 2015 to 2019, at a 600-bed tertiary care teaching hospital on patients who suffered from ACL tears and underwent ACL reconstruction. A control group of patients who underwent normal MRI with no pathologies was included for comparison.

The appropriate institutional review board (IRB) of the Jordan University Hospital (JUH) approved the proposal for this study. The Code of Ethics of the World Medical Association (Declaration of Helsinki) was followed while conducting the study. Informed written consent was obtained from the patients.

### Inclusion and exclusion criteria

The target population of this study consists of patients who were 18 years of age or above, had an ACL injury in one or both knees, performed their MRI imaging in the JUH database, and had their preoperative MRI available in the database. Exclusion criteria include patients younger than 18, had their MRI taken outside of JUH, were admitted for an ACL revision, or had suboptimal image quality, making it difficult to obtain the measurement accurately.

### Data collection process

The primary tool for data collection was the Jordan University Hospital’s Synapse (picture archiving and communication system) PACS system. Synapse was used to retrieve the MRI images that were analyzed using built-in measurement tools.

To prevent bias and data inaccuracies, specific definitions were given to all the 14 measurements, and two trained medical students were responsible for obtaining the measurements. Calculations were saved on each MRI image, and the final list was reviewed by a radiologist consultant and a senior radiology resident to ensure the proper method of obtaining the data and the accuracy of the measurements, as described below. To protect patients' privacy, measurement documentation was only done on the hospital premises, in the radiology department.

### MRI protocol

Two different MRI machines from the hospitals were used in this study. Patients were evaluated by a 1.5-Tesla and 3.0-Tesla MRI (Siemens Company, Munich, Germany). All MRI images were acquired with the patient placed in the supine position, the knee flexed to a 10-degree angle, and the inferior pole of the patella positioned in the center of the knee coil. A minimum of 20–30 slices was obtained for all planes, and the following planes were obtained: axial, coronal, and sagittal images, as the ACL is imaged optimally by using multiple planes to visualize the entire length of the ACL [21]. A slice thickness of 3 mm with a 0.4–1 mm interslice gap was used. The field of view was 14–16 × 15.1–17.2 cm, and the matrix size was 520–320 × 437–266.

### Obtaining the measurements on the MRI images

Using MRI imaging, 14 knee variables were studied in each participant. This included lateral meniscus (LM) posterior horn height, length, depth, and volume; medial meniscus (MM) posterior horn height, length, depth, and volume; lateral (LFC) and medial (MFC) femoral condyles sphere diameter; lateral (LTP) and medial (MTP) tibial plateau length; and patella tendon horizontal and vertical diameter.

**Fig. 1:**
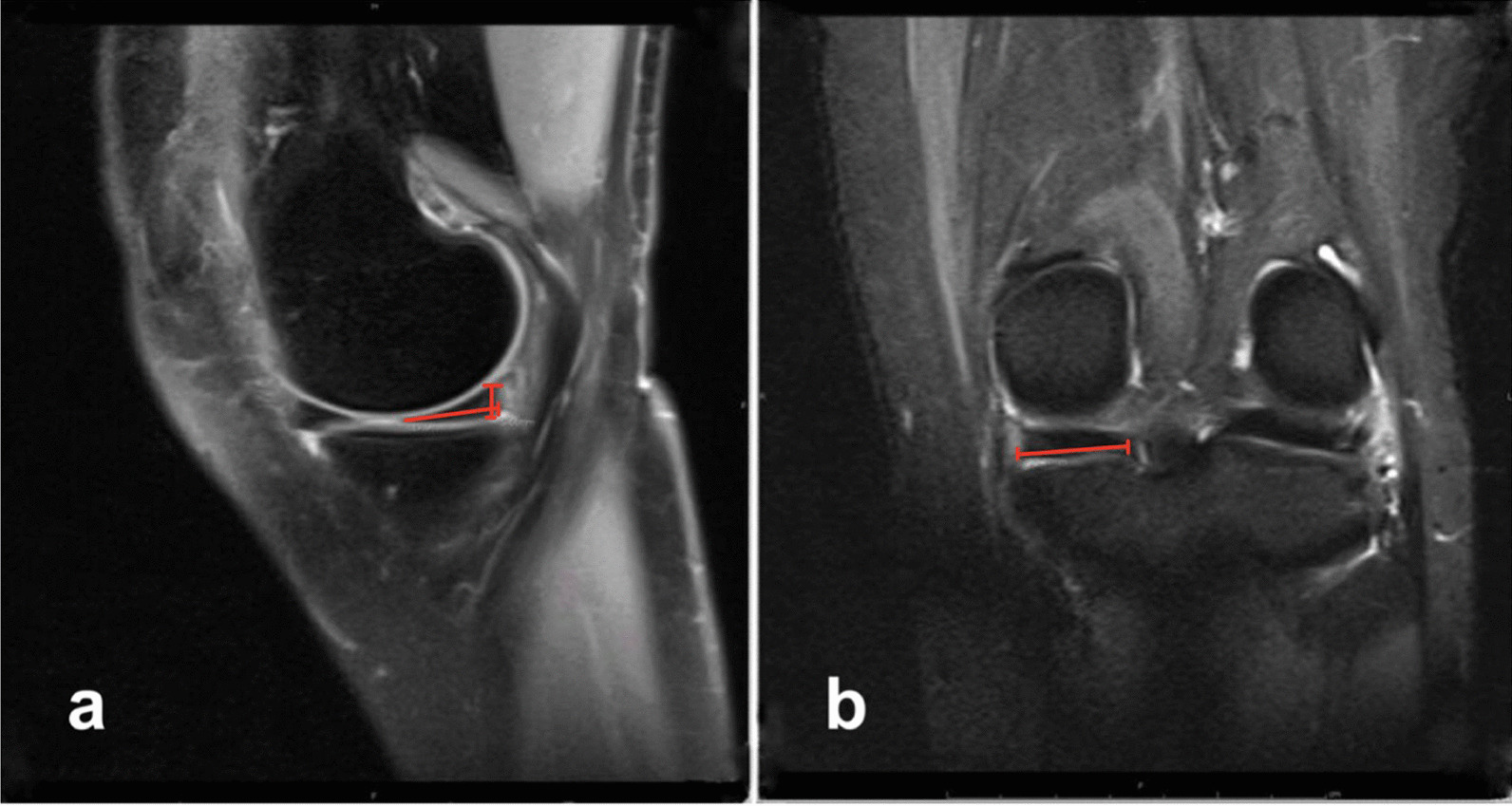
The measurement of medial meniscus posterior horn **a** height, **a** length, and **b** depth on a proton density sagittal magnetic resonance image

The LM and MM of each patient were measured using a sagittal plane, with a perpendicular line drawn down the vertical axis of the posterior horn of the medial or lateral meniscus, measuring its height, then a transverse line was drawn through the horizontal axis of the posterior horn of the medial or lateral meniscus measuring its length, guided by the corresponding first third of the section in the coronal view (Fig. 1a). For LM and MM depth, a transverse line was drawn through the horizontal axis of the posterior horn of the medial or lateral meniscus in the coronal plane, measuring its depth, guided by the corresponding middle of the section in the sagittal view (Fig. 1b). The meniscal volume was calculated by multiplying the height, length, and depth by 0.5.

**Fig. 2:**
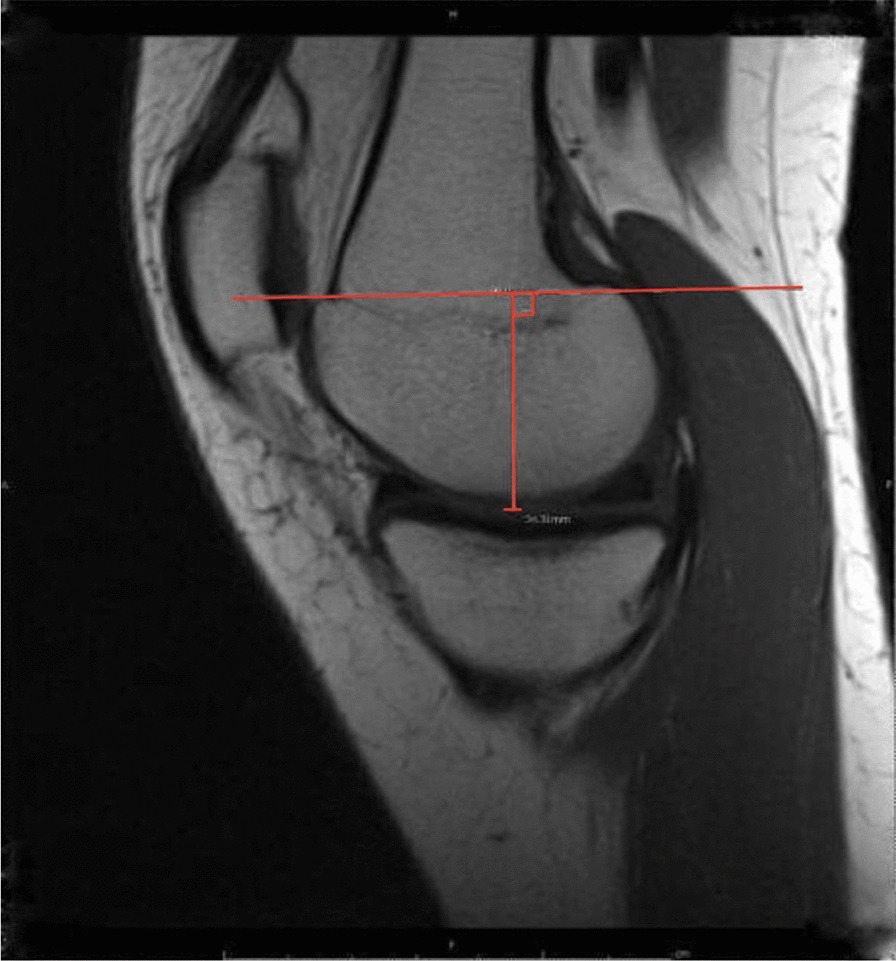
The measurement of medial femoral condyle sphere diameter on a T1 sagittal magnetic resonance images

The LFC and MFC were measured by using a sagittal plane, a transverse line drawn through the growth plate of the LFC or MFC, then a perpendicular line drawn down to the longest point on the horizontal axis of the medial or lateral meniscus, measuring the sphere diameter and guided by the corresponding middle of the section on the coronal view (Fig. 2).

**Fig. 3:**
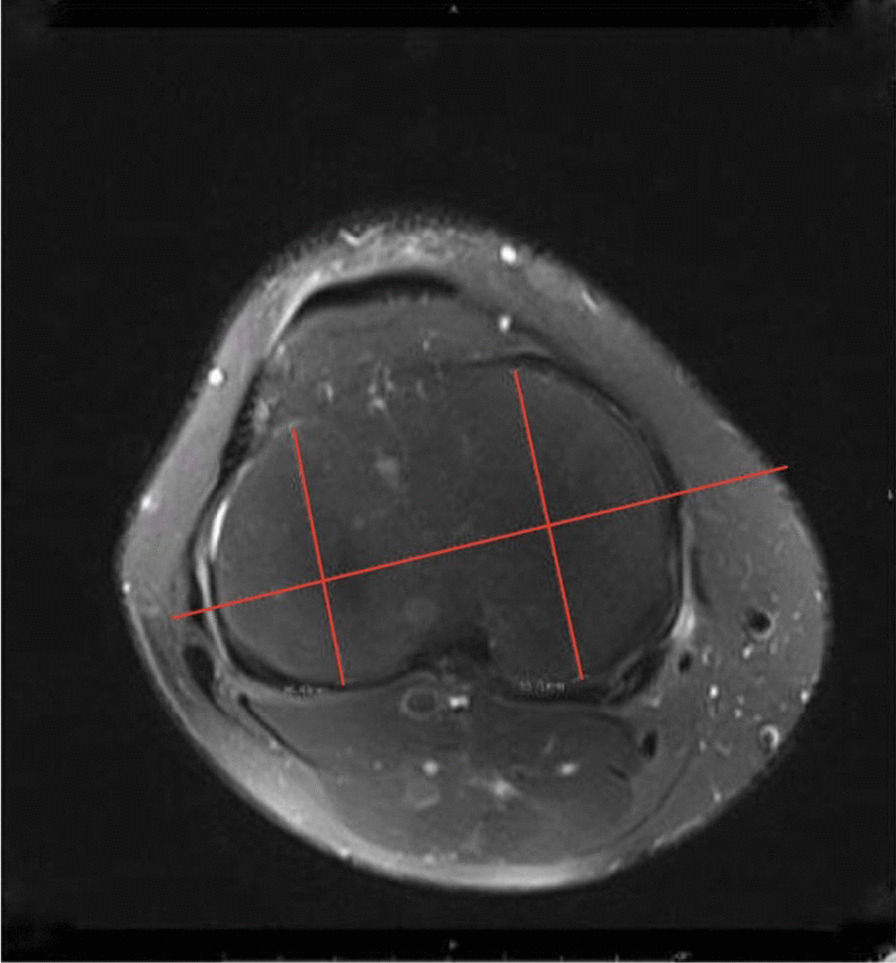
The measurement of medial tibial plateau length on a T2 axial view magnetic resonance image

The LTP and MTP were examined in an axial plane, a transverse line drawn through the proximal transtibial axis, extending from the medial edge of the medial plateau to the lateral margin of the lateral plateau (Fig. 3).

**Fig. 4:**
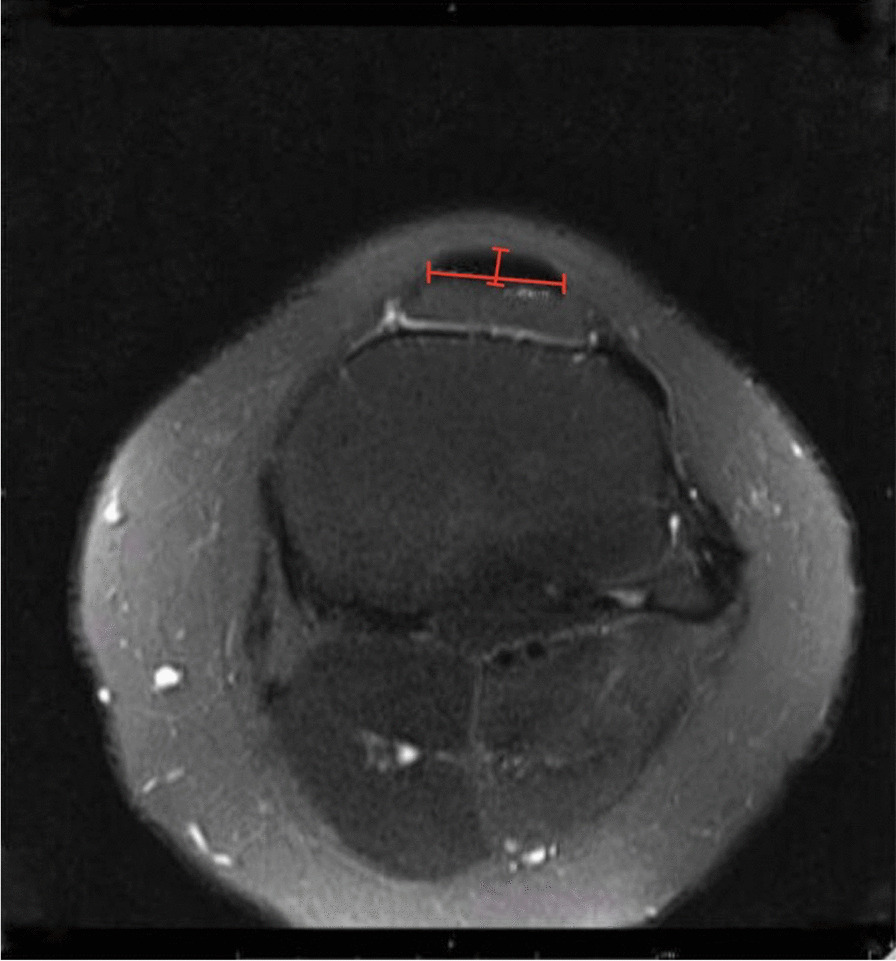
The measurement of patellar tendon horizontal and vertical length on a T2 axial view magnetic resonance image

The Patellar tendon horizontal and vertical diameters were measured from an axial, horizontal, and vertical line drawn through the patellar tendon, measuring its length and height, respectively, guided by the corresponding thickest point on the sagittal view (Fig. 4).

### Statistical analysis

SPSS version 28.0 (Chicago, USA) in our analysis. We used mean ± standard deviation to describe continuous variables (e.g. age). Count (frequency) to describe other nominal variables (e.g. gender). An independent sample t-test to analyze the mean difference between measurements and both cases and controls was performed and presented the data in mean (standard deviation). The effect size was calculated using Cohen's d. Binary logistic regression was used to identify the independent risk factors associated with ACL injury. For each significant independent sample t-test, the receiver operating characteristic curve (ROC) and the area under the curve (AUC) and its 95% CI were calculated. Statistical significance was defined as a *p*-value of less than 0.05.

## Results

In total, 85 patients were included in this study, and there were 60 (70.6%) men and 25 (29.4%) women; the age ranged from 18 to 49 years, with a mean age of 31.24 ± 9.81 years. The mean age among the cases was 27.87 ± 8.40 (range, 18–48) and in the control group was 37 ± 9.46 (range, 18–49). 54 (63.5%) had an ACL injury, while 31 (36.5%) were healthy controls. Table 1 shows a comparison between cases and controls with regard to age and gender.

**Table 1:** Demographics of the cases and controls

		Cases		Controls	
		Count (%)	Mean ± SD	Count (%)	Mean ± SD
Gender	F	7 (13.0%)		18 (58.1%)	
	M	47 (87.0%)		13 (41.9%)	
Age (years)			27.87 ± 8.40		37 ± 9.46

Our results showed a significant difference between ACL injury patients and controls in regard to LM posterior horn depth (*p* = 0.007) with a mean difference of 2.24 higher in the affected group (95% CI 0.62 to 3.86), MM posterior horn length (*p* = 0.01) with a mean difference of 2.53 higher in the controls (95% CI 0.62–4.44), MM volume (*p* = 0.007) with a mean difference of 424.06 higher in controls (95% CI 120.39–727.72), MFC sphere diameter (*p* = 0.004) with a mean difference of 2.39 higher in cases (95% CI 0.78–3.99), and patellar tendon horizontal diameter (*p* = 0.018) with a mean difference of 1.76 higher in cases (95% CI 0.31–3.21). Table 2 shows the details of the cases and control MRI knee measurements.

**Table 2:** Comparison of Knee MRI measurement between the cases and controls

	Cases		Controls		*p* value	Cohen's d effect size
	Mean	Standard deviation	Mean	Standard deviation		
LM posterior horn height	7.06	1.70	6.73	1.14	0.343	–
LM posterior horn length	11.02	2.79	11.41	2.88	0.539	–
LM posterior horn depth	25.64	3.69	23.4	3.25	0.007*	0.632 (0.169–1.092)
LM volume	987.38	350.87	880.75	476.24	0.241	–
MM posterior horn height	7.40	2.15	7.35	1.05	0.904	–
MM posterior horn length	14.97	4.42	17.5	3.55	0.01*	0.620 (0.147–1.088)
MM posterior horn depth	23.59	3.79	22.97	3.21	0.447	–
MM volume	1083.91	755.62	1507.97	511.26	0.007*	0.626 (0.172–1.076)
LFC sphere diameter	36.11	2.8	35.44	3.18	0.320	–
MFC sphere diameter	38.61	3.74	36.22	3.28	0.004*	0.667 (0.212–1.118)
LTP length	41.02	4.41	39.35	4.13	0.089	–
MTP length	49.54	3.69	49.43	6.99	0.935	–
Patella tendon horizontal diameter	27.03	3.08	25.27	3.41	0.018*	0.551 (0.095–1.004)
Patellar tendon vertical diameter	4.65	0.90	4.60	0.81	0.400	–

All measurements are in mm; asterisks indicate statistical significance (*p* < 0.05)

*LM* lateral meniscus, *MM* medial meniscus, *LFC* lateral femoral condyles, *MFC* medial femoral condyles, *LTP* lateral tibial plateau, *MTP* medial tibial plateau

Variables that showed potentially interesting associations with a level of significance (*p* < 0.05) from the univariate analysis were included in the multivariate logistic regression. Binary regression analysis revealed that LM posterior horn depth (OR = 1.27; 95% CI = 1.03–1.56; *p* = 0.028), MM posterior horn length (OR = 0.71; 95% CI = 0.55–0.93; *p* = 0.013), and MFC sphere diameter (OR = 1.20; 95% CI = 1.01–1.43; *p* = 0.035) were independent risk factors for ACL rupture (Table 3).

**Table 3:** Logistic regression association between MRI knee measurements and risk of ACL injury

Variables	AOR	95% Confidence interval	*p* Value
LM posterior horn depth	1.266	1.026–1.562	0.028
MM posterior horn length	0.714	0.547–0.931	0.013
MM volume	1	0.998–1.001	0.801
MFC sphere diameter	1.202	1.013–1.425	0.035
Patella tendon horizontal diameter	1.192	0.974–1.458	0.088

*LM* lateral meniscus, *MM* medial meniscus, *MFC* medial femoral condyles

**Fig. 5:**
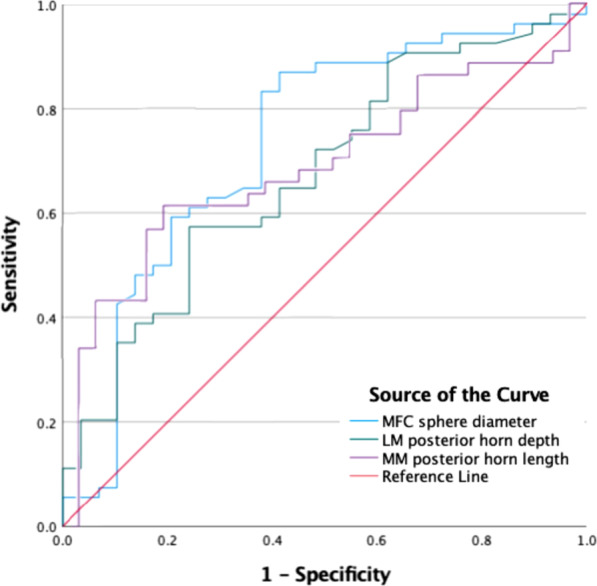
Receiver operating characteristic curve demonstrated the effects of the MFC sphere diameter, LM posterior horn depth, and patellar tendon horizontal length on ACL injury. Abbreviations: MFC, medial femoral condyles; LM, lateral meniscus

The ROC curve was used to determine the diagnostic accuracy and ability of the MRI knee measurement that was significant in the binary logistic regression. The highest AUC was reported for the ROC curve of MFC sphere diameter (0.747; 95% CI, 0.632–0.862; *p* < 0.001). Of the remaining significant measurements, the AUC of MM posterior horn length (0.686; 95% CI, 0.565–0.808; *p* = 0.006), and the AUC of LM posterior horn depth (0.675; 95% CI, 0.555–0.795; *p* = 0.009) (Fig. 5).

## Discussion

ACL injuries are the most common knee injuries [4], so identifying the risk factors and mechanics is an essential step in developing the basis of the preventive methods [22]. This is of extreme importance as these injuries can have serious consequences on the athlete’s career with a greatly increased risk of early osteoarthrosis and additional effects on their quality of life regardless of the management technique used [9]. Our data showed that the dimensions of certain knee structures are associated with the occurrence of an ACL injury. Increased LM posterior horn depth, MFC sphere diameter, and decreased medial meniscus posterior horn depth were found to be associated with ACL injury. In contrast, several variables, including LM posterior horn height, length, and volume; MM posterior horn height, length, and volume; LFC sphere diameter LTP, MTP, patella tendon horizontal, and vertical diameter, did not have a difference in mean between the two groups. To add to this, our regression model found that LM posterior horn depth had the highest significant predictive value for ACL rupture, followed by MFC sphere diameter and MM posterior horn length.

Similar studies have investigated different knee joint measurements and their relation to ACL injuries. In a case–control study identifying risk factors through MRI imaging, a narrower intercondylar notch was found to be associated with the risk of ACL rupture in a young population [23]. Park et al. found the notch width and medial condyle width to be significant risk factors for ACL injuries in males, and the notch width and medial condyle medial to lateral ration and notch width index in the female group [16]. In another case–control study, significant differences between the ACL group and the non-ACL group were found for bicondylar width, medial condyle width, and lateral condyle width [17]. Numerous associations with ACL injury have been described, including decreased intercondylar femoral notch size, decreased depth of concavity of the medial tibial plateau, increased tibial plateau slope, and increased anterior–posterior knee laxity [12]. However, the relationship between other knee measurements studied in this article and the risk of ACL injury has yet to be investigated.

In 1971, Lahlaïdi observed a mechanical connection between the posterior horn of the lateral meniscus and the ACL [24]. In 2010, Zemirline et al. confirmed these findings and found 13 of 14 dissected non-arthritic knees to have this connection, which they named the lateral meniscoligamentous band [25]. In a histological study, it was observed that mechanoreceptors in the structure also play a role in knee joint stability [26]. Despite this, the normal variation of this structure in individuals and the effect of how different knee dimensions may impact its role are not discussed in the literature. According to our results, there is a significant difference between ACL injury patients and controls in regard to LM posterior horn depth. Hence, considering that a deeper posterior horn of the lateral meniscus affects the location of the lateral meniscoligamentous band, it may contribute to decreased knee joint stability.

While studying distal femur morphometry, Murshed et al. noted that the size of the condyle has an impact on motor mechanics and that the size difference in the femoral condyle might be a risk factor by affecting knee joint rotation [27]. In our results, the medial femoral condyle diameter in ACL patients was found to be significantly different than those without. Nevertheless, our results showed no difference between the controls and cases in terms of the lateral femoral condyle. On the other hand, various studies have found that a smaller lateral femoral condyle causes greater knee laxity, which places these individuals at a greater risk of ACL injury [18, 19, 28]. This discrepancy could have resulted from the different male to female ratio in our study versus the studies mentioned, since males are known to have a larger lateral femoral condyle [27].

Suprasanna et al. found that the wider patellar tendon-tibial shaft angle to be a risk factor for noncontact ACL injury, such that it can itself be an independent predictor according to the binary regression model [20]. This is speculated to be the result of the increase in patellar tendon tibial shaft angle to elongating the ACL, resulting in an increase in anterior shear force on the tibia [29]. In our study, a different measurement of the patella, the patella tendon horizontal diameter, had a significant difference in the mean between the cases and controls. Furthermore, our results show that both medial and lateral tibial plateau length are not statistically different between the ACL and non-ACL groups. An imaging-based case–control study found similar results, that no parameters of the tibial plateau were a risk factor for ACL injury [30]. Nonetheless, there was no consensus on whether a larger or shallower slope causes even amongst studies that associated tibial plateau parameters with ACL injury. In their study, Hashemi et al. found that a shallow tibial plateau is a risk factor for an ACL injury. However, Rahnemai-Azar et al. and Grassi et al. found that an increased tibial plateau slope predisposes to an ACL injury [31–33]. Further research should be done on the slope and length of the tibial plateau and how they are linked to ACL injuries.

The results should be interpreted with caution, owing to several limitations. First, it is limited by the small sample size. Moreover, all patients included in the current study were from a single-center and thus may be subject to regional ethnic sampling biases. Another limitation is that the cases and controls were not controlled for age or gender. However, our study had several strengths. It’s the first in-depth study to investigate these knee MRI measurements between the ACL-injured and non-injured ACL subjects. The study used a precise definition with a clearly defined protocol for all the measurements, which can be very easily reproduced. Therefore, we believe that our findings reflect a very important finding that provides a rationale for future clinical studies. We encourage that future studies use propensity score matching to minimize any bias that may exist between cases and controls.

## Conclusion

The study provides preliminary evidence that there might be differences in the dimensions of specific knee structures between ACL-injured and non-injured subjects, which most likely act in combination with several other risk factors to influence the risk of ACL injury. However, a comprehensive multivariate risk model that considers all the internal and external risk factors in combination should be investigated in order to develop proper prevention techniques and appropriate health care and counseling for those who may be at increased risk of suffering multiple injuries. Future studies should be done to allow precise stratification of patients and define a cutoff value to allow for the quantification of the risk of an ACL rupture in MRI. This can help the doctor with patient counseling, prevention, and changes to the postoperative rehabilitation protocol.

## Data Availability

The datasets generated during and/or analyzed during the current study are not publicly available due to regulations in the University of Jordan Research Ethics Committee, but may be made available from the corresponding author on reasonable request.

## Abbreviations

ACL: Anterior cruciate ligament
MRI: Magnetic resonance imaging
JUH: Jordan University Hospital
LM: Lateral meniscus
MM: Medial meniscus
LFC: Lateral femoral condyles
MFC: Medial femoral condyles
LTP: Lateral tibial plateau
MTP: Medial tibial plateau
ROC: Receiver operating characteristic curve
AUC: Area under the curve
